# Supplementary material for: Association between hematocrit and the 30-day mortality of patients with sepsis: A retrospective analysis based on the large-scale clinical database MIMIC-IV
Source: PLoS One. 2022 Mar 24;17(3):e0265758. doi: 10.1371/journal.pone.0265758 (PMC8947025; doi:10.1371/journal.pone.0265758)
Supplement: Attachment — Submitted filename: review PLOS-ONE 1.doc [file pone.0265758.s001.doc]

Dear Editor,

Thank you for the opportunity to review the manuscript “Association between hematocrit and the 30-day mortality of patients with sepsis: a retrospective analysis based on the large-scale clinical database MIMIC-IV”, paper code PONE-D-21-35992.

The authors presented a large-scale retrospective analysis on the association between hematocrit and mortality in patients with sepsis.

The study has some limitations that are all addressed by the authors. Accepting these limitations, the authors show the interesting results useful for scientific discussion.

Hereby I recommend to reconsider this work after minor revision.

My best regards.

Looking forward to work with you again,

Valentina Bellini

Comments for authors:

Dear authors,

You have presented a large-scale retrospective analysis on the association between hematocrit and mortality in patients with sepsis.

The topic is interesting and it would be important to find a real contribution in this setting.

Please give your statement to the following points:

**1. Abstract**

The abstract is clear enough.

**2. Introduction**

- The introduction was repeated twice; delete the second repetition.

**3. Materials and Methods**

- It has not been included if a sample size estimation has been carried out; has it been calculated?

- It is not clear enough to me whether the selected patients were already diagnosed with sepsis upon entering the ICU.

**4.Results**

- In the figure 1, please add legend.

**5. Discussion**

- It is written that "The HCT levels obtained in our research were all information of patients with sepsis on the first day after entering the ICU, which suppressed the progression of the disease and changes in laboratory parameter values induced by fluid resuscitation therapy"; but the patients who had already been diagnosed with sepsis upon admission to the ICU could not have already started fluid resuscitation therapy? Or could not they have been more fragile patients already in fluid overload?

- Please specify better the clinical message that the authors want to send.

**6. Conclusion**

- In my opinion the words "hematocrit during ICU" is misleading; better to specify “hematocrit in the first 24 hours after ICU admission”.

**7. Tables**

- Table 2 appears to be repeated; please check the data.

- Tables 1 and 2 lack legends; please enter.

**8. References**

Please check the journal’s guidelines

It would be necessary to revisit the English language.

Best regards
