# Supplementary material for: Association between hematocrit and the 30-day mortality of patients with sepsis: A retrospective analysis based on the large-scale clinical database MIMIC-IV
Source: PLoS One. 2022 Mar 24;17(3):e0265758. doi: 10.1371/journal.pone.0265758 (PMC8947025; doi:10.1371/journal.pone.0265758)
Supplement: Attachment — Submitted filename: Response to Reviewers.docx [file pone.0265758.s002.docx]

Dear editors and reviewers:

Thanks for your letter and the reviwers’comments about our manuscript entitled “Association between hematocrit and the 30-day mortality of patients with sepsis: a retrospective analysis based on the large-scale clinical database MIMIC-IV”.All these suggestions are valuable for me to revise my manuscript. We have studied comments carefully and have made a correction which we hope meet with approval.

Suggestions from editors:

The topic is interesting and it would be important to find a real contribution in this setting.

Please give your statement to the following points:

1. Abstract

The abstract is clear enough.

2. Introduction

- The introduction was repeated twice; delete the second repetition.

3. Materials and Methods

- It has not been included if a sample size estimation has been carried out; has it been calculated?

- It is not clear enough to me whether the selected patients were already diagnosed with sepsis upon entering the ICU.

4.Results

- In the figure 1, please add legend.

5. Discussion

- It is written that "The HCT levels obtained in our research were all information of patients with sepsis on the first day after entering the ICU, which suppressed the progression of the disease and changes in laboratory parameter values induced by fluid resuscitation therapy"; but the patients who had already been diagnosed with sepsis upon admission to the ICU could not have already started fluid resuscitation therapy? Or could not they have been more fragile patients already in fluid overload?

- Please specify better the clinical message that the authors want to send.

6. Conclusion

- In my opinion the words "hematocrit during ICU" is misleading; better to specify “hematocrit in the first 24 hours after ICU admission”.

7. Tables

- Table 2 appears to be repeated; please check the data.

- Tables 1 and 2 lack legends; please enter.

8. References

Please check the journal’s guidelines

It would be necessary to revisit the English language.

Dear editors,the revised portion are marked in red in our paper.

**Responses to Reviwers:**

Reviewer #1

**1. -Introduction paragraph (line 81-93) seems repeated**

Answer:We are very sorry for our neligence of this paragraph.And then the repetitive sentence in the introductory paragraph has been deleted.

**2. -reporting checklist is missing, I suggest to use the RECORD guidelines (The REporting of studies Conducted using Observational Routinely-collected health Data ) Please refer to http://journals.plos.org/plosmedicine/article?id=10.1371/journal.pmed.100188-**

Answer:Considering the reviwer’s suggestion,we have made some modifications based on the guidelines.

**3.-Please indicate in detail how do you selected the sepsis population in the MIMIC database. Did you used the "concepts" script for mimic-iv in order to improve the reproducibility of the analysis? (Johnson, A. E., Stone, D. J., Celi, L. A., & Pollard, T. J. (2018). The MIMIC Code Repository: enabling reproducibility in critical care research. Journal of the American Medical Informatics Association : JAMIA, 25(1), 32–39. or an extraction based on ICD-9 codes (Epidemiology of severe sepsis in the United States: analysis of incidence, outcome, and associated costs of care. Angus DC, Linde-Zwirble WT, Lidicker J, Clermont G, Carcillo J, Pinsky MRCrit Care Med. 2001 Jul; 29(7):1303-10.) and Martin et al. (The epidemiology of sepsis in the United States from 1979 through 2000. Martin GS, Mannino DM, Eaton S, Moss M**

**N Engl J Med. 2003 Apr 17; 348(16):1546-54)**

Answer:Firstly,we download the zip package of the database from the official website for a series of installation, and then enter the interface of PostgreSQL 6.0. We use **SELECT*FROM** statement to find the icd-9 code of sepsis in **d_icd_diagnoses,** and then use **SELECT WHERE** statement to find the related icd-9 code of sepsis in **diagnoses_icd** (99591/99592)We used the "concepts" script for mimic-iv in order to improve the reproducibility of the analysis.

**4.-Line 119-120 report the use of non parametric test, please specify the distribution of data**

Answer:Considering the reviewer’s suggestion the data distribution has been shown in Table 1.

Reviewer #2

**1. Abstract**

The abstract is clear enough

Dear Reviewer,thank you very much for the recognition.

**2.Introduction**

**- The introduction was repeated twice; delete the second repetition.**

Answer:Thank you for your careful review.The repetitive sentence in the introductory paragraph has been deleted.

**3.Materials and Methods**

**- It has not been included if a sample size estimation has been carried out; has it been calculated?**

**- It is not clear enough to me whether the selected patients were already diagnosed with sepsis upon entering the ICU.**

Answer:Thank you for your careful review.This sample size has been calculated.The data of sepsis patients we present in the MIMIC IV database by SQL statements are diagnosed on admission to the ICU.This can be determined by referring to the r

elevant details of this database on the official website.(https://physionet.org/content/mimiciv/0.4/).

**4.Results**

**- In the figure 1, please add legend.**

Answer:Thank you for your careful review.We have added the legend.(Details can be found in the revised version)

**5.Discussion**

**- It is written that "The HCT levels obtained in our research were all information of patients with sepsis on the first day after entering the ICU, which suppressed the progression of the disease and changes in laboratory parameter values induced by fluid resuscitation therapy"; but the patients who had already been diagnosed with sepsis upon admission to the ICU could not have already started fluid resuscitation therapy? Or could not they have been more fragile patients already in fluid overload?**

Answer:Thank you for the reviewer's suggestion, We have reworked it and marked in red.(Line-138-139)

**6Conclusion**

**- In my opinion the words "hematocrit during ICU" is misleading; better to specify “hematocrit in the first 24 hours after ICU admission”.**

Answer:Thank you for the reviewer's suggestion, I have adopted your revision and made the statement changes.(Line-151)

**7.Tables**

**- Table 2 appears to be repeated; please check the data.**

**- Tables 1 and 2 lack legends; please enter.**

Answer:The table has been made in detail。（Details can be found in the revised version）

**8.References**

**Please check the journal’s guidelines**

It would be necessary to revisit the English language.

Changes have been made to the reference specification.We have made revisions to the language and punctuation.Please point out again if there are any shortcomings.

Thank you for your careful review. We really appreciate your efforts in reviewing our manuscript during this unprecedented and challenging time. We wish good health to you, your family, and community. Your careful review has helped to make our study clearer and more comprehensive.
